# The teachings of Long COVID

**DOI:** 10.1038/s43856-021-00016-0

**Published:** 2021-07-12

**Authors:** Nisreen A. Alwan

**Affiliations:** 1 School of Primary Care, Population Sciences and Medical Education, Faculty of Medicine, University of Southampton, Southampton, UK; 2 NIHR Southampton Biomedical Research Centre, University of Southampton and University Hospital Southampton NHS Foundation Trust, Southampton, UK; 3 NIHR Applied Research Collaboration Wessex, Southampton, UK

**Keywords:** Signs and symptoms, Health services, Epidemiology

## Abstract

Alwan discusses the lessons learnt over the past year regarding Long COVID, prolonged illness resulting from SARS-CoV-2 infection, and their implications for public health policy and disease management, drawing insight form her own lived experience, research, and advocacy work with Long COVID.

## Teaser

Long COVID is prolonged illness resulting from SARS-CoV-2 infection. Its serious implications for individuals and society have been missing from public communication and pandemic policy. Here, I draw on my lived experience, research, and advocacy work with Long COVID and reflect on the lessons learnt.

Long COVID is the state of not recovering several weeks following acute infection with SARS-CoV-2, whether tested or not^1^. It is a patient-made^2^ umbrella term for this condition, which may involve multiple pathologies. The underlying mechanisms are still largely unknown, but hypotheses include inflammatory or autoimmune processes, organ damage and scarring, hypercoagulability, endothelial damage, or even persistent viral protein in the body^3,4^. Based on the UK Office for National Statistics (ONS) estimates, the prevalence of Long COVID is around 1 in 7 people at three months from the infection, and it is most common in working-age adults, but also occurs in other age groups, including children^5^. More recent ONS figures indicate that there are 376,000 people in the UK who have had Long COVID for at least one year^6^. It has a wide range of symptoms, but the most common are exhaustion, breathlessness, muscle aches, cognitive dysfunction, including poor memory and difficulty concentrating, headache, palpitations, dizziness and chest tightness or heaviness. The nature of the symptoms is mostly relapsing, resulting in significant dysfunction and limitations in a relatively large proportion of sufferers^6,7^.

It seemed nobody had thought about the enormous implications of chronic disease as a result of letting SARS-CoV-2 spread through the community, assuming it would all be fine for those classed as non-vulnerable.

During the past year, I have been advocating for Long COVID, as well as doing research on it. I experienced it after developing COVID-19 symptoms in March 2020. My acute illness was not severe, so I did not go to hospital, as the medical advice at the time was to isolate at home, and that, like flu-like illness, one would be completely recovered within a week or two. This also meant I did not have access to testing to confirm infection, as community testing stopped in the UK on March 12, 2020^8^. Although I felt improvements, the illness did not go away after several weeks. Some of my symptoms, the chest heaviness, muscle aches, and fatigue, remained fluctuating for months, while new symptoms, such as palpitations, also appeared. Every time I felt it was almost over, symptoms came back. I started recognising and avoiding some of the activities that triggered the symptoms, but I could not always work out what caused the relapses.

The constant cycle of disappointment at not completely recovering was devastating. The never-ending symptoms and their effect on my daily activities were a cause for worry. It was somewhat reassuring that so many others were posting similar stories on social media, but it was a struggle to get Long COVID recognised by governments and national health agencies as a serious problem back then. It seemed nobody had thought about the enormous implications of chronic disease as a result of letting SARS-CoV-2 spread through the community, assuming it would all be fine for those classed as non-vulnerable. As I wrote in a previous piece ‘death is not the only thing to count in this pandemic, we must count lives changed’^9^. I urged public health agencies to quantify and define Long COVID^10,11^. Over the last year, I have been engaging in forums to raise awareness on its significance, impact, and scale. I have also worked with other people living with Long COVID to research the characteristics of the illness^7^. Through this journey, I have learnt some lessons that apply not only to Long COVID but more widely to pandemic preparedness, equality, and social justice, and how medicine and society deal with similar chronic conditions.

The first lesson was how much our understanding, as scientists or physicians, can be enriched by patient experience. This includes genuine patient involvement in all stages of science and healthcare design, but may also include us wearing the two hats of patient and expert. Unfortunately, a lot of healthcare professionals and health scientists across the world caught SARS-CoV-2 with many suffering the consequences of Long COVID. In the UK, 3.6% of all healthcare staff were estimated to have Long COVID^5^. The experience of the illness not only brings deep understanding and appreciation of its real-life impact, but also of the questions that need answering^12^. People with lived experience must have a central role in shaping the research and services agenda because they are experts in living with the disease. Even with substantial patient involvement in shaping care and research, some sections of society will always have more representation in decision-making forums than others. Therefore, without seeking insight and input from those usually unheard, our response will always be inadequate.

Another lesson was that we need systems in place that measure morbidity in addition to mortality. We have always been better at measuring the acute over the chronic, but it is the latter that has the most long-lasting impact on societies. At the beginning of the pandemic, long-term illness and ensuing disability due to COVID were completely dismissed and did not shape policy decisions. This is partly because they were not adequately quantified, and the models informing policy and public opinion used short-term outcomes of hospitalisation and death. It is disheartening to still frequently see recovery confused with short-term survival or hospital discharge. We need systems to record recovery and continued illness following infection, accurately and universally. Disease registers have been employed for other chronic conditions such as cancer and could prove very valuable for Long COVID as well as other post-viral illnesses.

A third lesson was that we must challenge stereotyped narratives that tend to dominate the Long COVID discourse. Long COVID has been predominately pictured as something that mainly ails middle-aged women. However, the difference in the prevalence between women and men seems relatively small (15% vs 13% according to ONS estimates^5^). Women have experienced not being believed about their symptoms with other similar chronic conditions, such as chronic fatigue syndrome and fibromyalgia^13^. This has the potential to lead to stigma and institutional discrimination^14^. When the dismissal of concerns and symptoms by service providers and employers is compounded by demographic, ethnic, social, and economic pre-existing structural disparities, the injustice is exacerbated. The stigma can become internalised potentially depriving people with lived experience of Long Covid from recognition, support, and services because they do not want to face the dismissal, disbelief, and denial. We must not repeat past mistakes of stereotyping and pushing those already disadvantaged away from seeking help.

To avoid the effect of stereotyping, stigma, and variation in recognition, and to measure the effect of Long COVID on systems, the economy, and the whole of society, we need to agree case definitions as soon as possible. Science on the topic is evolving and case definitions will need to be frequently updated, but we cannot afford to wait. People living with Long COVID need proper clinical assessments, medical investigations, and a diagnosis. A diagnosis is necessary not only for treatment and rehabilitation purposes, but also to maintain livelihoods. Without it, people with what are considered ‘unexplained symptoms’ may lose out on employment rights and benefits, leading to financial hardship that can exacerbate their illness. The diagnosis could simply be an umbrella term like Long COVID that encompasses some uncertainty about how it manifests. A case definition for research can be more stringent than that for the purpose of surveillance. Criteria used for clinical diagnosis must be the most inclusive because people’s lives depend on them (11). The case definitions must be based on clinical assessment and not be dependent on laboratory tests, since there is a range of problems with these, including access, affordability, and accuracy.

Though perhaps the most important lesson that Long COVID taught me, and I hope it can teach others, is that showing humility in the face of uncertainty is the first right step to deal with a phenomenon that we do not fully understand. Throughout the pandemic, I have seen uncertainty in science, medicine and public health communicated with certainty. This has been largely damaging, and that includes the case of Long COVID. The possibility that COVID-19 might not be a short illness for all, was entirely dismissed from public communication, despite multiple examples of devastating long-lasting effects of other viruses. Assumptions have been made about the nature, cause, and mode of treatment of Long COVID, despite a lack of evidence to support them. Acknowledging we do not know everything does not mean inaction. It means informed action with honesty, which may involve applying the precautionary principle until we know more.

The pandemic is not over, and it is peaking in many parts of the world. Therefore, preventing Long COVID should be high on everyone’s agenda. Long COVID messaging must be incorporated in all prevention policies including vaccination and non-pharmacological interventions. The effect of COVID-19 vaccines in modifying the course of Long COVID is still uncertain and under investigation^15^. In the meantime, the primary purpose of vaccination in relation to Long COVID should be to prevent it in those who do not have it, and to prevent re-infection in those who do.

As for me, I am grateful that my Long COVID has been a lighter guest in 2021, with less frequent and shorter visits. This is sadly not the story of everybody who is living with it, with many not improving, or deteriorating over time. Let us, for their sake, not repeat past mistakes and learn from the global experience of this phenomenon to help all people living with similar under-researched chronic conditions, and prevent more from happening.
